# A Simple Score Scale Composed of Serum Inflammatory Factors Assists in Psoriasis Arthritis Prediction among Patients with Psoriasis Vulgaris

**DOI:** 10.3390/biomedicines12092130

**Published:** 2024-09-19

**Authors:** Wanrong Huang, Yao Li, Yuanyuan Xu, Rui Gao, Long Geng

**Affiliations:** 1 Department of Dermatology, The First Hospital of China Medical University, Shenyang 110001, China; hwr1998@163.com (W.H.);; 2 National Joint Engineering Research Center for Theranostics of Immunological Skin Diseases, Shenyang 110001, China; 3 Key Laboratory of Immunodermatology, Ministry of Education and NHC, Shenyang 110001, China; 4 The Reproductive Medical Center, Department of Obstetrics and Gynecology, West China Second University Hospital, Sichuan University, Chengdu 610041, China; kaojui@yeah.net; 5 Key Laboratory of Birth Defects and Related Diseases of Women and Children, Sichuan University, Ministry of Education, Chengdu 610041, China

**Keywords:** serum inflammatory indicators, psoriatic arthritis, psoriasis vulgaris, risk recognition, score scale

## Abstract

**Aim:** To compare the levels of serum inflammatory indicators in psoriasis vulgaris patients who progress to PsA and those not, as well as to establish and validate a simple score scale for predicting PsA for psoriasis vulgaris patients. **Methods:** A cross-sectional study was performed at a university hospital in China to recruit five hundred and seventy-seven patients who had been diagnosed with psoriasis vulgaris for at least 10 years. After evaluation, 86 were enrolled in the PsA group, and the others were selected as the control group. Eight serum inflammatory factors were detected and compared between the two groups. A simple score scale for PsA prediction was established and validated. **Results:** Serum CRP, IL-6, and TNF-α levels were significantly higher in the PsA group than in the control group. A simple score scale composed of CRP, IL-6, and TNF-α was established. The sensitivity was 59.30% and the specificity was 83.50% for predicting PsA among all psoriasis vulgaris patients when the cut-off value of the total score was set as 1.8 points. The simple score scale presented a predictive value for progressing to PsA among all psoriasis vulgaris patients internally (AUC = 0.788), and the performance was also conformed in psoriasis vulgaris patients receiving topical treatment (AUC = 0.746), systemic treatment (AUC = 0.747) and biological treatment (AUC = 0.808), respectively. The predictive performance of this scale was also validated by an external retrospective cohort (AUC = 0.686). **Conclusions:** CRP, IL-6, and TNF-α were potential indicators to recognize PsA risk in patients with psoriasis vulgaris. A simple score scale may provide new insights for early prediction of PsA among psoriasis vulgaris patients.

## 1. Introduction

Psoriasis is an immune-mediated, inflammatory disease that affects both the skin and joints. As with other chronic dermatoses, it may cause various types of disfiguration and disability [1]. One of the most common clinical manifestations of psoriasis is demarcated erythematous plaques covered by thick silver scales; approximately 90% of patients complained of this manifestation, and we defined them as plaque-type psoriasis or psoriasis vulgaris [2]. Psoriatic arthritis (PsA) is not only a severe complication of psoriasis vulgaris, but also a progressive inflammatory musculoskeletal disease leading to joint damage and disability [3]. The prevalence of PsA worldwide is 133 per 100,000 [4] and the proportion in psoriasis patients is approximately 30% [3]. As reported, psoriasis patients have a higher risk of developing PsA than others, and the average duration from psoriasis to PsA is 10 years [5]. The progression from psoriasis vulgaris to PsA is irreversible and has no specific treatment [6,7]; even a six-month diagnostic delay can lead to more severe joint damage and a worse prognosis [8,9]. Timely diagnosis and treatment are important for improving the prognosis and reducing the disability rate of PsA patients.

However, early diagnosis of PsA for psoriasis vulgaris patients is currently very difficult. The widely used Classification for Psoriatic Arthritis (CASPAR) criteria based on clinical manifestations, emphasized the evidence of psoriasis histories, the negative results of rheumatic factor, and image findings [10]. It mainly meant that classification might not be adequate to diagnose PsA at an early stage [11]. Exploring the effective serological markers of PsA for psoriasis vulgaris patients is a valuable topic. According to previous studies, some genetic, proteomic, and metabolic markers were shown to be associated with a higher risk of PsA among psoriasis vulgaris patients [12]. Serum inflammatory indicators are a group of factors derived from serum to estimate body inflammation, including various proteins, peptides, and cytokines. Studies found that some serum inflammatory indicators took part in the pathogenesis of PsA, but whether these indicators could predict PsA risk among psoriasis vulgaris patients was unclear [13,14].

In this study, we designed a cross-sectional study to compare the levels of serum inflammatory indicators between psoriasis vulgaris patients who progressed to PsA and those who did not, and establish a simple score composed of serum inflammatory factors for predicting PsA for psoriasis vulgaris patients. The score was validated by a retrospective cohort, thus providing more references for early recognition of PsA.

## 2. Materials and Methods

### 2.1. Study Design

Firstly, a cross-sectional study was performed in the Department of Dermatology, the First Hospital of China Medical University from January 2022 to December 2023. The socio-demographic information, treatment information and serum samples of psoriasis vulgaris patients were collected, and eight inflammatory indicators that were reported to take part in the pathogenesis of PsA, including C reactive protein (CRP), interleukin-2 (IL-2), interleukin-4 (IL-4), interleukin-6 (IL-6), interleukin-10 (IL-10), interleukin-17A (IL-17A), tumor necrosis factor-α (TNF-α) and interferon-γ (IFN-γ), were detected by an electrochemiluminescence immunoassay platform (cobas E411, Roche Diagnostics, Indianapolis, IN, USA). A simple score scale for predicting PsA risk among psoriasis vulgaris patients was established and internally validated. Secondly, a retrospective cohort involving psoriasis vulgaris patients who detected these above indicators between January 2016 and December 2019 was collected in the Department of Dermatology, the First Hospital of China Medical University, and these patients were all followed up until 31 December 2023. This retrospective cohort was used to validate the performance of the established score scale externally. This study was approved by the institution’s ethics committee (protocol code 2024-692), and all participants had signed informed consent.

### 2.2. Participants and Groups

In the cross-sectional study, we recruited psoriasis vulgaris patients who satisfied the inclusion criteria established by all authors, including (1) having typical lesions: demarcated erythematous plaques covered by thick silver scales; (2) being diagnosed with psoriasis vulgaris by skin biopsy or by an experienced dermatologist; (3) age more than 18 years old; (4) without other confirmed rheumatologic or musculoskeletal disease (osteoarthritis, infectious arthritis, rheumatoid arthritis, reactive arthritis, coercive spondylitis, etc.); and (5) without any malignant tumor. All participants were divided into the PsA group and the control group, according to whether they were diagnosed as PsA or not. The diagnosis of PsA was referred to the CASPAR criteria [10]. In brief, the CASPAR criteria were composed of 7 terms with total of 8 points, including current psoriasis (2 points), personal history of psoriasis (1 point), family history of psoriasis (1 point), typical psoriatic nail dystrophy (1 point), rheumatoid factor negative (1 point), current dactylitis (1 point) and juxta-articular new bone formation on hand or foot X-ray (1 point). The total number of points ≥ 3 was regarded as PsA diagnosis.

In the retrospective cohort, we selected psoriasis vulgaris patients with the same inclusion criteria as the above, but only patients who were not yet diagnosed as PsA and in whom we had detected the serum levels of CRP, IL-6, and TNF-α were included in the end. The serum levels of CRP, IL-6 and TNF-α were detected for baseline assessment before the start of systemic or biological treatment. Thus, the levels of peripheral inflammatory indicators were less affected by systemic and biological treatment in this retrospective cohort. All participants in the retrospective cohort were divided into the high-score group and the low-score group, according to the established simple score scale.

### 2.3. Information Collection in Cross-Sectional Study

While participants were included, two authors reviewed the medical records independently. Baseline information was collected by the Electronic Medical Records System, including gender, age, height, weight, BMI, disease duration of psoriasis vulgaris, the severity of lesions (body surface area, BSA), smoking status, family history of psoriasis vulgaris, nail involvement, diabetes, hypertension, dactylitis, and image evidence of joint damage. Besides this, white blood cell (WBC), neutrophil (NE), lymphocyte (LY), monocyte (MONO), D-dimer (D-D), alanine aminotransferase (ALT), aspartate aminotransferase (AST), albumin (ALB), serum creatinine (Scr), C3, C4, IgG, IgA, IgM, IgE, CD3^+^ T cell, CD4^+^ T cell, CD8^+^ T cell, CD45^+^ lymphocyte, erythrocyte sedimentation rate (ESR) and rheumatoid arthritis (RF) in peripheral blood were also collected. The treatment status of all participants was collected in this system. Because the clinical treatment of psoriasis vulgaris and PsA is complicated, we can only divide the treatment status into topical treatment, systemic treatment and biological treatment, to decrease the potential influence of different treatment background on results. Topical treatment refers to patients receiving only topical medicines on inclusion; systemic treatment refers to patients who received methotrexate, acitretin or ciclosporin; biological treatment refers to patients receiving various biological, but no traditional, immunosuppressors. At the same time, we collected the peripheral blood samples for all participants by a red-head blood collection tube with coagulant (BD Biosciences, Franklin Lakes, NJ, USA), and acquired serum samples by centrifugation for 3000 rpm, 15 min. The serum samples were stored at −80 °C until detection. Serum inflammatory indicators of CRP, IL-2, IL-4, IL-6, IL-10, IL-17A, TNF-α, and IFN-γ were detected by an electrochemiluminescence immunoassay platform (cobas E411, Roche Diagnostics), and the results were determined as negative or positive, according to the reference interval at that time.

### 2.4. Information Collection and Follow-Up in the Retrospective Cohort

When a psoriasis vulgaris patient was enrolled into the retrospective cohort, we collected his/her baseline information through the Electronic Medical Records System. The information includes gender, age, height, weight, BMI, disease duration of psoriasis vulgaris, the severity of lesions (body surface area, BSA), smoking status, family history of psoriasis vulgaris, diabetes, and hypertension. Psoriasis vulgaris patients who had been diagnosed as PsA were excluded from the cohort. Of course, the levels and category (negative/positive) of serum CPR, IL-6, and TNF-α were recorded. We followed up on these included patients by phone or social media, and focused on the items in the CASPAR criteria to determine whether and when they progress to PsA (outcome); the terminal date of follow-up was set as 31 December 2023. If the patients were lost to follow-up because of rejection, death, or other reasons, they were excluded from this cohort subsequently. The authors did not know the results of the serum inflammatory indicators before follow-up.

### 2.5. The Simple Score Establishment and Validation

In the cross-sectional study, we compared the serum inflammatory indicators between the PsA group and the control group by univariable analysis. The serum inflammatory indicators that were significantly different between the two groups were used for establishing a simple score by stepwise logistic regression analysis. To simplify, we calculated the adjusted odds ratio (OR) and corresponding 95% confidence interval (CI), and the OR was then transferred as a point for every indicator. The total points were calculated and were used for predicting the PsA risk among psoriasis vulgaris patients.

#### 2.5.1. Internal Validation

The receiver operating characteristic (ROC) curve was drawn to calculate the area under the curve (AUC), and the Youden index was used to determine the suggested cut-off value. At the cut-off value, the discriminative accuracy was reported in terms of sensitivity and specificity. The internal validations were performed for all psoriasis vulgaris patients and patients in different treatment subgroups.

#### 2.5.2. External Validation

An external validation was performed in the retrospective cohort to estimate the performance of this simple score in predicting PsA risk for psoriasis vulgaris patients. The total points of the simple scores were calculated for every patient, and were divided into the high-score group or the low-score group, according to the scores ≥the cut-off value or less than the cut-off value, respectively. The prevalence of those progressing into PsA was compared among psoriasis vulgaris patients in the high-score group or the low-score group by using Kaplan–Meier curves and log-rank estimates, and the ROC curves, AUC, sensitivity, and specificity were used to estimate the performances of this simple score scale.

### 2.6. Statistical Analyses

Missing data in this study were imputed using the mean filling method (if missing data <5%). Continuous variables with a normal distribution were expressed as mean ± standard deviation (SD) and were compared by the Student’s *t*-test. Continuous variables with an abnormal distribution were expressed as medians (interquartile range) and were compared by the Mann–Whitney U test. Categorial variables were expressed as numbers (percentiles) and compared by the χ^2^ test. A value of *p* < 0.05 was considered to be significant for all statistical analyses. Data were analyzed by using SPSS software version 23.0 (IBM, Armonk, NY, USA) and MedCalc software version 19.4 (Ostend, Belgium).

## 3. Results

### 3.1. Baseline Information of the Cross-Sectional Study

Five hundred and seventy-seven psoriasis vulgaris patients were recruited in the cross-sectional study, and 86 of them were additionally diagnosed as PsA. The gender distribution of the two groups was similar (*p* = 0.935). There was no difference in age, BMI and disease duration between the two groups. There was a statistical difference in nail involvement (20% vs. 75.6%, *p* < 0.001) between the two groups. The PsA group had a higher prevalence of nail involvement than the control group (75.6% vs. 20.0%, *p* < 0.001). In the PsA group, there were 13 patients (15.1%) who had dactylitis and 73 patients (84.9%) who had image evidence of joint damage. Among the laboratory tests in medical records, lymphocyte number [1.76 (1.34–2.10) vs. 1.38 (1.09–1.68), *p* < 0.001] and D-dimer [0.56 (0.37–0.74) vs. 0.44 (0.27–0.76), *p* = 0.031] in the control group was higher than the PsA group, but other indicators were similar between the two groups. It is necessary to mention that more patients in the PsA group received systemic or biological treatments on inclusion, and patients in the control groups had a higher proportion of topical treatment (*p* = 0.008). The detailed baseline information of the two groups is shown in Table 1.

### 3.2. Serum Inflammatory Indicator Comparison

Among the serum inflammatory indicators, there were statistically significant differences in CRP, IL-2, IL-6, IL-17A, TNF-α, and IFN-γ between the two groups, but there was no statistical difference between IL-4 and IL-10. Moreover, we classified these serum inflammatory indicators into binary variables based on their normal range (CRP ≤ 6.00 mg/L, IL-2 ≤ 5.71 pg/mL, IL-4 ≤ 3.0 pg/mL, IL-6 ≤ 5.30 pg/mL, IL-10 ≤ 4.91 pg/mL, IL-17A ≤ 20.60 pg/mL, TNF-α ≤ 4.60 pg/mL, IFN-γ ≤ 7.42 pg/mL). The prevalence of positive CRP (37.1% vs. 62.8%, *p* < 0.001), IL-2 (8.1% vs. 1.2%, *p* = 0.020), IL-6 (6.5% vs. 43.0%, *p* < 0.001), TNF-α (29.1% vs. 47.7%, *p* = 0.001), and IFN-γ (15.9% vs. 27.9%, *p* = 0.007) were higher in the PsA group than the control group. The detailed information is shown in Table 2.

The serum inflammatory indicators that showed statistical differences were included in multivariable logistic regression analysis. To eliminate confounding factors, these indicators were all adjusted by gender, age, duration, BMI, smoking status, BSA, treatment status, LY, and D-D, respectively. The results indicated that CRP, IL-2, IL-6, IL-17A and TNF-α differ statistically with adjusted ORs (95%CI) (Table 3), while IFN-γ showed no statistical difference between two groups. The category serum inflammatory indicators were included in multivariable logistic regression analysis, simultaneously. Results suggested that there were statistical differences in category CRP, category IL-2, category IL-6, and category TNF-α, between the two groups. The detailed results are shown in Table 3.

### 3.3. A Simple Score-Scale Establishment

According to the results of multivariable regression analysis, category CRP, category IL-2, category IL-6, and category TNF-α were included in a logistic regression. The results indicated that CRP, IL-6, and TNF-α still differ statistically with adjusted ORs (adjusted p value) of 2.64 (*p* < 0.001), 10.73 (*p* < 0.001), and 3.36 (*p* < 0.001), while there was no statistical significance for IL-2 between the two groups after logistic regression analysis. After selection of variables, finally, we calculated the weight of these factors. If the result exceeds the reference value, it is positive. We assigned 1 point to positive CRP, 4 points to positive IL-6, and 1.3 points to positive TNF-α. The total number of points on the scale is 6.3. All participants were graded again, according to the score scale. The results and scale are in Table 4.

### 3.4. Internal Validation of the Simple Score Scale

**Figure 1:**
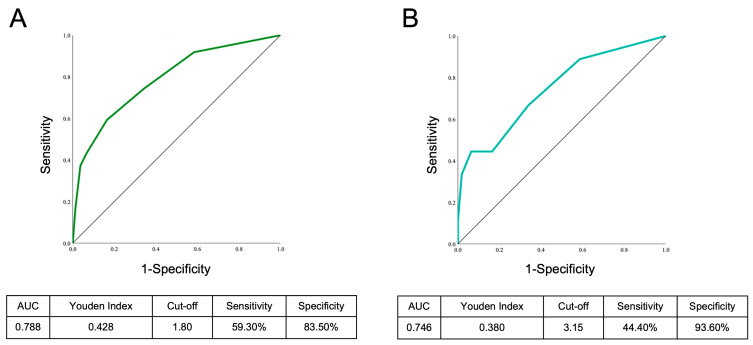

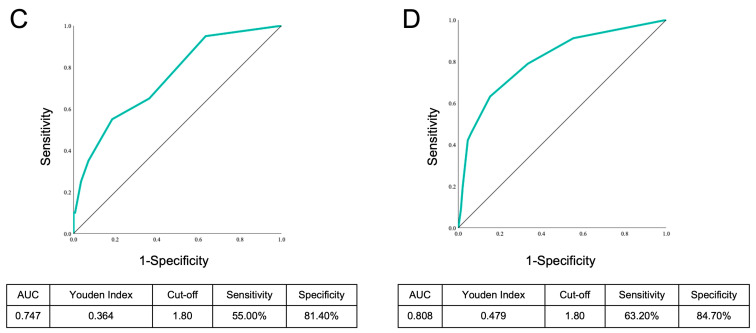
The ROC curve of the simple score in predicting PsA risk in the cross-sectional study. The ROC curve of the simple score in predicting PsA risk among all participants (**A**), the subgroup of participants who received topical treatment (**B**), the subgroup of participants who received systemic treatment (**C**) and the subgroup of participants who received biological treatment (**D**).

We established the ROC curves of the scale and calculated the corresponding AUC for all participants. The ROC curves revealed this rating scale had eligible discrimination (AUC = 0.788) to recognize PsA in psoriasis vulgaris patients. When the Youden index was 0.428, a sensitivity of 59.30% and a specificity of 83.50% with a cut-off value of 1.8 were observed. Patients with psoriasis vulgaris who achieved the threshold would be regarded as high risk for progressing to PsA according to this scale. The ROC curve is shown in Figure 1A. Moreover, to investigate the influence of different treatment statuses on the performance of this scale, we established the ROC curves and calculated the AUC for patients who received topical treatment, systemic treatment, and biological treatment respectively. The AUC was 0.746, 0.747, and 0.808 in the three treatment subgroups respectively. As for the cut-off value, we found that in the topical-treatment subgroup, the suitable cut-off value was 3.15 points, but in both the systemic- and biological-treatment subgroups, the suitable cut-off value was 1.80 points. These results are shown in Figure 1B–D.

### 3.5. External Validation of the Simple Score Scale

A total of 131 patients with psoriasis vulgaris but not PsA were enrolled in this retrospective cohort, according to the inclusion and exclusion criteria. On enrollment, only topical treatment was used for these patients. During the follow-up, 45 patients were lost because of rejection (n = 41) or death (n = 4). Among the 86 patients who were finally included in this retrospective cohort, the median follow-up period was 36 months, and 22 patients were diagnosed as PsA during the follow-up period. According to the simple score established before, 37 patients were classified into the high-score group and 59 patients were classified into the low-score group. There was no statistical difference in age, duration, BMI, smoking status, family history, diabetes, hypertension, and BSA between the two groups. However, the high-score group had higher proportions of positive CRP (63.0% vs. 32.2%, *p* = 0.007), IL-6 (70.4% vs. 0.0%, *p* < 0.001), and TNF-α (59.3% vs. 40.7%, *p* = 0.109) than the low-score group. The above results are shown in Table 5.

**Figure 2:**
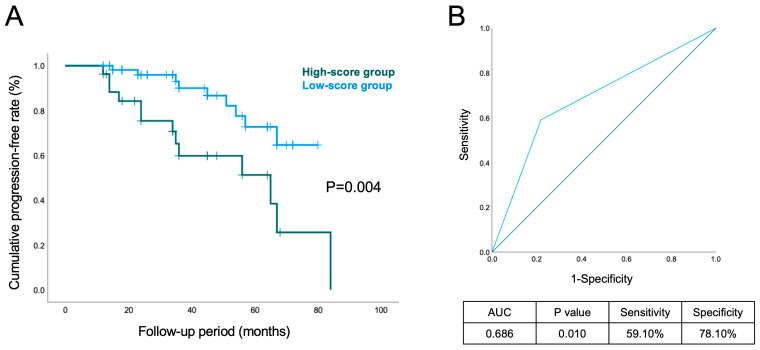
The results of external validation in a retrospective cohort. (**A**) Kaplan–Meier curves and log-rank estimates for the progression to PsA among psoriasis vulgaris patients with high and low scores. (**B**) The ROC curve of the simple score in predicting PsA risk in the retrospective cohort.

Moreover, the Kaplan–Meier curve and log-rank estimations showed that the high-score group had a significantly higher cumulative risk for suffering PsA than the low-score group (*p* = 0.001) (Figure 2A). The simple score, composed of CRP, IL-6 and TNF-α, with the cut-off value of 1.8, also showed satisfying predictive performance for PsA among the retrospective cohort of psoriasis vulgaris patients externally (AUC = 0.686, *p* = 0.010, sensitivity = 59.1%, specificity = 78.1%) (Figure 2B).

## 4. Discussion

PsA is not only a chronic inflammatory musculoskeletal disease but is also regarded as a severe complication of psoriasis vulgaris [15]. There were some similarities and differences between the pathogenesis of PsA and psoriasis vulgaris. For example, they are all associated with class I MHC alleles, while studies found that HLA-C*06 is a major risk factor for psoriasis vulgaris but not for PsA [3]. Despite the controversy in the mechanism, epidemiological studies indicated that PsA is more frequent in psoriasis vulgaris patients, and approximately 30% of patients with psoriasis vulgaris may develop PsA during their course of illness [16,17]. Thus, focusing on the risk factors of developing PsA and recognizing PsA as early as possible in psoriasis vulgaris patients is very important. However, the current CASPAR criteria were classification criteria based on clinical manifestations, which aimed to distinguish PsA from other rheumatic articular diseases, but did not provide a method for early diagnosis or even prediction of PsA [10]. There is no established serological marker or serological tool in PsA diagnosis specifically, at present. For these reasons, we designed this cross-sectional study and found that a simple score composed of inflammatory factors CRP, IL-6, and TNF-α had satisfying diagnostic and predictive effects on PsA among psoriasis vulgaris patients, as well as validating the performance externally, in a retrospective cohort. Since these inflammatory factors were easy to detect and non-invasive, we think this score provided a simple strategy for early prediction of PsA among psoriasis vulgaris patients.

In addition to our study, some previous studies also explored the risk factors of how psoriasis transits to PsA. Studies showed that environmental factors such as infection, trauma, smoking, occupation, and familial predisposition were associated with the development of PsA, but the link is weak and conflicting [18,19,20,21]. Studies also suggested that genetic susceptibility, obesity, nail involvement, and psoriasis severity were risk indicators for developing PsA in psoriasis vulgaris patients [22,23,24,25]. However, these factors had unsatisfying predictive performances for suffering PsA among those suffering from psoriasis vulgaris [26]. Serological indicators presented a good prospect for predicting many diseases, and were well investigated in autoimmune disorders such as rheumatoid arthritis. A negative RF is considered the only serological indicator for diagnosing, but not for predicting, PsA, according to the CASPAR classification criteria [10]. The predictive performance of other serological indicators in PsA was rarely explored.

Inflammatory indicators were shown to take part in the pathogenesis of PsA. CRP is an acute-phase protein with good stability and accuracy [27]. It is a non-specific marker of inflammation and tissue injury. During the inflammatory process, CRP was produced by hepatic cells and rapidly elevated in peripheral blood under the stimulation of proinflammatory cytokines such as IL-1ß, IL-6, IL-17, and TNF-α. However, CRP was elevated in various conditions such as malignant carcinoma or infective disease, so using CRP to detect PsA singly was not precise enough [28]. IL-6, a proinflammatory cytokine produced by lymphoid cells, was proved to be increased in synovial tissue and serum of PsA patients [29]. It activated fibroblast-like synovial cells and B cells, which activated STAT3 signaling to enhance proinflammatory cytokine production [30]. IL-6 gene expression was higher in patients with PsA than in patients who only had psoriasis skin lesions, and it was closely related to joint disorders [31]. TNF-α was also a proinflammatory cytokine produced mainly by macrophages and monocytes, which participated in the Th17 signaling pathway and played an important role in the formation of skin lesions and synovial damage [30,32]. It was critical for the initiation, development and severity of psoriasis, as well as the transition to PsA [33,34], and thus TNF-α inhibitors were used in the clinical treatment of PsA [35,36]. Moreover, other cytokines such as IL-2, IL-4, IL-10, IL-17, IL-22 and IFN-γ were proven to be involved in PsA pathogenesis, and some of them were detected to be elevated in synovial fluid but not peripheral blood [3]. In this study, the cross-sectional study identified the fact that serum CRP, IL-2, IL-6, TNF-α and IFN-γ levels were significantly increased in PsA patients, and positive serum CRP, IL-6 and TNF-α were independently associated with PsA among psoriasis vulgaris patients.

We innovatively established a simple score scale composed of these three significant inflammatory factors based on the cross-sectional data, and validated the performance for recognizing PsA internally. The sensitivity of 59.3% and the specificity of 83.5% were calculated to show a good performance in recognizing PsA. The disruptive effect of treatment status on results were also considered, because we all know that systemic treatment and biological treatment may decrease the levels of inflammatory indicators, significantly. Thus, we adjusted the confusing variable of treatment status on inflammatory indicators during indicator selection by multivariable regression analysis, and divided all patients into three different subgroups, to detect, accordingly, the performance of this simple score scale separately. Interestingly, the scale has acceptable discriminative accuracies in all subgroups, but has different suitable cut-off values. For patients receiving topical treatment, the cut-off value was set as 3.15 points, but for patients receiving systemic treatment and biological treatment, the cut-off value was set as 1.80 points. It may be explained that patients receiving systemic and biological treatment had decreased inflammatory indicators levels compared to those who received topical treatment. It also indicated that this scale can be used for PsA risk estimation in psoriasis vulgaris patients with different treatment backgrounds, after the adjustment of cut-off values.

However, based on the cross-sectional design, we did not obtain information on the causal relationship between inflammatory indicators and PsA. We established a retrospective cohort to validate the simple score scale externally. In the retrospective cohort, the previous results of serum CRP, IL-6, and TNF-α detection were collected for the included psoriasis vulgaris patients who did not progress into PsA, and the clinical prognosis was followed up for at least 4 years. We also found that the simple score scale had good performance for prospectively predicting the PsA risk among psoriasis vulgaris patients. According to the above results, we think that detecting serum CRP, IL-6, and TNF-α levels for psoriasis vulgaris patients may help clinicians evaluate the risk for progressing into PsA; psoriasis vulgaris patients with high simple scores should be more focused on their arthritis manifestations and should be exanimated more frequently. According to our knowledge, this was the first study that established a score scale by serum inflammatory indicators for predicting the PsA risk for psoriasis vulgaris patients, and it provided a new idea for both researchers and clinicians focusing on this problem.

There were some limitations to this study. Firstly, this score scale may only be applied to chronic plaque psoriasis, which is psoriasis vulgaris. Considering the large number of patients and stability of clinical manifestation, we only included psoriasis vulgaris patients in our study. Special variants of psoriasis, such as generalized pustular psoriasis, may show different systematic inflammatory reactions from plaque psoriasis. Secondly, because of the restriction of the Electronic Medical Records System, as well as the fact that the clinical treatment of psoriasis vulgaris is very complicated, we did not obtain the detailed information on their medications. We divided participants into topical, systemic, and biological treatment, robustly, to decrease the influence of medicines on the results, but the difference between different medicines was not explored. The cut-off value of this score scale for patients with different treatment backgrounds should be investigated in the future. Thirdly, only a small sample size was used in both the cross-sectional study and the retrospective cohort. As for the technological restriction, only a few psoriasis vulgaris patients showed serum CRP, IL-6 and TNF-α detected previously, so most patients could not be included in this retrospective cohort. The small sample size restricted the generalizability of our study. Last, but not least, the period for psoriasis vulgaris patients progressing to PsA is very long; thus, the follow-up period in our study was not enough to cover every possible patient. Based on these above limitations, we emphasized the fact that the results of our study must be interpreted with caution, and, when using the simple score scale, establishing a cut-off value that is suitable for the local environment is also very important. Establishing a large sample size, a multicenter approach, and a large prospective psoriasis vulgaris cohort in the future may provide a higher level of clinical evidence on the association between inflammatory indicators and PsA, as well as optimizing the existing score.

## 5. Conclusions

We conducted a cross-sectional study to compare the levels of serum inflammatory indicators between psoriasis vulgaris patients who progressed to PsA and those who did not, and found that CRP, IL6 and TNF-α levels were all higher in PsA patients than in controls. Thus, we established a simple score scale composed of serum CRP, IL-6 and TNF-α levels to estimate the risk for progressing to PsA among psoriasis vulgaris patients, and validated the scale internally and for a retrospective cohort. The simple score scale showed good performances internally and externally. We provided new insight into the association between serum inflammatory factors and PsA risk for psoriasis vulgaris patients and designed a simple tool to help clinicians estimate the PsA risk for psoriasis vulgaris patients more conveniently. Future studies with a multi-center and prospective cohort design should be performed to validate the clinical value of this simple score. Further classification of psoriasis vulgaris patients according to treatments should be considered in the future, to improve the effects of this scale.

## Figures and Tables

**Table 1:** Baseline information and laboratory examinations of participants in cross-sectional study.

	Total (n = 577)	Control Group (n = 491)	PsA Group (n = 86)	*p* Value
Gender [n(%)]				0.935
Male	313 (54.2)	266 (54.2)	47 (54.7)	
Female	264 (45.8)	225 (45.8)	39 (45.3)	
Age (years)	43 (32–55)	43 (32–54)	43.5 (30–57.25)	0.659
Duration (years)	11 (6–20)	11 (6–20)	12.5 (6–21)	0.547
Height (m)	1.68 (1.62–1.75)	1.68 (1.62–1.75)	1.68 (1.61–1.74)	0.668
Weight (kg)	61 (55–68)	61 (55–68)	60 (56–68)	0.637
BMI (kg/m^2^)	21.56 (19.36–23.88)	21.55 (19.26–23.99)	21.59 (19.87–23.39)	0.978
Smoker [n(%)]				0.155
Yes	91 (15.8)	73 (14.9)	18 (20.9)	
No	486 (84.2)	418 (85.1)	68 (79.1)	
Family history [n(%)]				0.314
Yes	153 (26.5)	134 (27.3)	19 (22.1)	
No	424 (73.5)	357 (72.7)	67 (77.9)	
BSA	7 (4–10)	7 (4–10)	7 (4–11)	0.185
Diabetes [n(%)]				0.202
Yes	70 (12.1)	56 (11.4)	14 (16.3)	
No	507 (87.9)	435 (88.6)	72 (83.7)	
Hypertension [n(%)]				0.171
Yes	92 (15.9)	74 (15.1)	18 (20.9)	
No	485 (84.1)	417 (84.9)	68 (79.1)	
Nail involvement [n(%)]				<0.001
Yes	163 (28.2)	98 (20.0)	65 (75.6)	
No	414 (71.8)	491 (80.0)	21 (24.4)	
Dactylitis [n(%)]				-
Yes	-	-	13 (15.1)	
No	-	-	73 (84.9)	
Image evidence [n(%)]				-
Yes	-	-	31 (36.0)	
No	-	-	55 (64.0)	
Treatment status [n(%)]				0.008
Topical treatment	118 (20.5)	109 (22.2)	9 (10.4)	
Systemic treatment	160 (27.7)	140 (28.5)	20 (23.3)	
Biological	299 (51.8)	242 (49.3)	57 (66.3)	
WBC (109/L)	7.45 (6.32–8.83)	7.43 (6.34–8.79)	7.53 (5.85–9.31)	0.968
NE (109/L)	5.01 (4.21–6.34)	4.95 (4.23–6.24)	5.18 (3.96–7.19)	0.483
LY (109/L)	1.68 (1.31–2.07)	1.76 (1.34–2.10)	1.38 (1.09–1.68)	<0.001
MONO (109/L)	0.53 (0.37–0.66)	0.52 (0.36–0.66)	0.54 (0.39–0.64)	0.521
D-D (mg/L)	0.55 (0.35–0.74)	0.56 (0.37–0.74)	0.44 (0.27–0.76)	0.031
ALT (U/L)	17 (14–20)	17 (14–19)	18 (13–23.25)	0.107
AST (U/L)	20 (15–25)	20 (16–24)	18.5 (12.0–28.0)	0.097
ALB (g/L)	39.6 (35.3–42.6)	39.8 (35.6–42.6)	37.6 (33.0–43.3)	0.097
Scr (μmol/L)	59 (51–68)	59 (52–68)	56 (47–68.25)	0.307
C3 (g/L)	1.12 (0.93–1.32)	1.12 (0.93–1.33)	1.11 (0.93–1.27)	0.409
C4 (g/L)	0.35 (0.28–0.44)	0.36(0.28–0.44)	0.33 (0.27–0.40)	0.079
IgG (g/L)	11.30 (9.78–12.72)	11.27 (9.74–12.67)	11.46 (9.96–13.19)	0.137
IgA (g/L)	2.58 (1.94–3.20)	2.57 (1.93–3.20)	2.63 (2.10–3.32)	0.348
IgM (g/L)	1.00 (0.74–1.29)	1.00 (0.75–1.27)	0.98 (0.67–1.35)	0.918
IgE (g/L)	142 (77–244)	139 (77–233)	166 (72.5–277.25)	0.889
CD3 (cells/μL)	1390 (1116–1645)	1410 (1123–1658)	1335 (1025–1528)	0.091
CD4 (cells/μL)	784 (612–954)	793 (628–955)	736 (568–947.25)	0.204
CD8 (cells/μL)	580 (450–725)	583 (451–724)	568 (450–757.5)	0.646
CD45+ (cells/μL)	1685 (1256–2049)	1663 (1222–2043)	1789 (1408.25–2084)	0.147
ESR (mm/h)	15 (9–23)	16 (9–23)	13 (5.75–26.25)	0.051
RF (IU/mL)	7.4 (5.9–8.9)	7.3 (5.8–8.9)	7.70 (6.68–9.03)	0.093

PsA, psoriasis arthritis; BMI, body mass index; BSA, body surface area; WBC, white blood cell; NE, neutrophil; LY, lymphocyte; MONO, monocyte (MONO); D-D, D-dimer; ALT, alanine aminotransferase; AST, aspartate aminotransferase; ALB, albumin; Scr, serum creatinine; ESR, erythrocyte sedimentation rate; RF, rheumatoid arthritis. *p* < 0.05 was regarded as statistically different.

**Table 2:** The differences in serum inflammatory indicators between the two groups.

	Total (n = 577)	Control Group (n = 491)	PsA Group (n = 86)	*p* Value
CRP (mg/L)	4.3 (2.6–12.0)	4.0 (2.5–9.4)	12.0 (3.0–21.5)	<0.001
CRP [n(%)]				<0.001
Positive	236 (40.9)	182 (37.1)	54 (62.8)	
Negative	341 (59.1)	309 (62.9)	32 (37.2)	
IL-2 (pg/mL)	2.50 (1.58–3.64)	2.53 (1.62–3.72)	2.40 (1.10–3.36)	0.013
IL-2 [n(%)]				0.020
Positive	41 (7.1)	40 (8.1)	1 (1.2)	
Negative	536 (92.6)	451 (91.9)	85 (98.8)	
IL-4 (pg/mL)	2.04 (1.28–3.41)	2.00 (1.24–3.60)	2.31 (1.49–3.09)	0.113
IL-4 [n(%)]				0.434
Positive	168 (29.1)	146 (29.7)	22 (25.6)	
Negative	409 (70.9)	345 (70.3)	64 (74.4)	
IL-6 (pg/mL)	2.34 (1.23–3.58)	2.30 (1.23–3.30)	3.42 (1.50–12.97)	<0.001
IL-6 [n(%)]				<0.001
Positive	69 (12.0)	32 (6.5)	37 (43.0)	
Negative	508 (88.0)	459 (93.5)	49 (57.0)	
IL-10 (pg/mL)	2.75 (1.66–4.88)	2.76 (1.65–4.89)	2.71 (1.68–4.85)	0.844
IL-10 [n(%)]				0.971
Positive	140 (24.3)	119 (24.2)	21 (24.4)	
Negative	437 (75.7)	372 (75.8)	65 (75.6)	
IL-17A (pg/mL)	4.13 (2.36–7.97)	3.89 (2.15–7.23)	6.60 (3.58–21.82)	<0.001
IL-17A [n(%)]				0.467
Positive	3 (0.5)	3 (0.6)	0(0.0)	
Negative	574(99.5)	488 (99.4)	86(100.0)	
TNF-α (pg/mL)	2.96 (1.76–6.29)	2.82 (1.65–6.13)	4.50 (2.44–7.68)	<0.001
TNF-α [n(%)]				0.001
Positive	184(31.9)	143(29.1)	41 (47.7)	
Negative	393(68.1)	348 (70.9)	45 (52.3)	
IFN-γ (pg/mL)	2.34 (1.18–4.06)	2.18 (1.09–3.48)	3.43 (1.92–8.12)	<0.001
IFN-γ [n(%)]				0.007
Positive	102 (17.7)	78 (15.9)	24 (27.9)	
Negative	475 (82.3)	413 (84.1)	62 (72.1)	

CRP, C reactive protein; IL-2, interleukin-2; IL-4, interleukin-4; IL-6, interleukin-6; IL-10, interleukin-10; IL-17A, interleukin-17A; TNF-α, tumor necrosis factor-α; IFN-γ, interferon-γ. *p* < 0.05 was regarded as statistically different.

**Table 3:** Adjusted results of serum inflammatory indicators by univariable logistic regression.

	Adjusted OR	Adjusted 95% CI	Adjusted *p* Value
CRP	1.044	1.026–1.063	<0.001
Positive CRP	2.885	1.753–4.748	<0.001
IL-2	0.817	0.703–0.950	0.009
Positive IL-2	0.106	0.014–0.794	0.029
IL-6	1.269	1.173–1.372	<0.001
Positive IL-6	11.144	6.004–20.684	<0.001
IL-17A	1.030	1.014–1.045	<0.001
Positive IL-17A	<0.001	0.000–0.001	0.999
TNF-α	1.038	1.010–1.067	0.008
Positive TNF-α	1.932	1.181–3.161	0.009
IFN-γ	1.008	0.973–1.046	0.647
Positive IFN-γ	1.588	0.901–2.798	0.110

Adjusted for gender, age, disease duration, BMI, smoking status, BSA, LY, and D-D, respectively.

**Table 4:** Score scale of the transition from psoriasis vulgaris to PsA.

	Adjusted OR	Adjusted 95% CI	Adjusted *p* Value	Weight
Positive CRP	2.64	1.56–4.47	<0.001	1
Positive IL-2	0.15	0.02–1.13	0.066	-
Positive IL-6	10.73	5.89–19.56	<0.001	4
Positive TNF-α	3.36	1.95–5.78	<0.001	1.3

CRP, C reactive protein; IL-2, interleukin-2; IL-6, interleukin-6; TNF-α, tumor necrosis factor-α. *p* < 0.05 was regarded as statistically different.

**Table 5:** Baseline information and follow-up results of participants in the external retrospective cohort.

	Total (n = 86)	High-Score Group (n = 37)	Low-Score Group (n = 59)	*p* Value
Gender [n(%)]				0.468
Male	46 (53.5)	16 (59.3)	30 (50.8)	
Female	40 (46.5)	11 (40.7)	29 (49.2)	
Age (years)	47 (42–57)	46 (37–67)	47 (42–56)	0.830
Duration (years)	5 (8–11)	8 (4–14)	8 (5–11)	0.730
Height (m)	1.67 (1.62–1.72)	1.68 (1.62–1.78)	1.67 (1.62–1.72)	0.259
Weight (kg)	64 (58–70)	66 (60–73)	63 (56–69)	0.220
BMI (kg/m^2^)	22.95 (20.79–24.79)	22.79 (21.55–24.92)	23.03 (20.72–24.78)	0.867
Smoker [n(%)]				0.114
Yes	31 (36.0)	13 (48.1)	18 (30.5)	
No	55 (64.0)	14 (51.9)	41 (69.5)	
Family history [n(%)]				0.344
Yes	20 (23.3)	8 (29.6)	12 (20.3)	
No	66 (76.7)	19 (70.4)	47 (79.7)	
BSA	6 (5–7)	6 (5–8)	6 (5–7)	0.966
Diabetes [n(%)]				0.959
Yes	29 (33.7)	9 (33.3)	20 (33.9)	
No	57 (66.3)	18 (66.7)	39 (66.1)	
Hypertension [n(%)]				0.877
Yes	34 (39.5)	11 (40.7)	23 (39.0)	
No	52 (60.4)	16 (59.3)	36 (61.0)	
Simple scores	1.3 (1.0–2.3)	4.0 (2.3–5.3)	1.0 (0.0–1.3)	<0.001
Positive CRP [n(%)]	36 (41.9)	17 (63.0)	19 (32.2)	0.007
Positive IL-6 [n(%)]	19 (22.1)	19 (70.4)	0 (0.0)	<0.001
Positive TNF-α [n(%)]	40 (46.5)	16 (59.3)	24 (40.7)	0.109
Follow-up period (months)	36 (21–56)	34 (18–56)	36 (23–57)	0.493
PsA [n(%)]	22 (25.6)	13 (48.1)	9 (15.3)	0.001

PsA, psoriasis arthritis; BMI, body mass index; BSA, body surface area; CRP, C reactive protein; IL-6, interleukin-6; TNF-α, tumor necrosis factor-α. *p* < 0.05 was regarded as statistically different.

## Data Availability

The raw data supporting the conclusions of this article will be made available by the authors on request.

## Abbreviations

PsA	psoriatic arthritis
CASPAR	Classification for Psoriatic Arthritis
CRP	C-reactive protein
IL	interleukin
BMI	body mass index
BSA	body surface area
WBC	white blood cell
NE	neutrophil
LY	lymphocyte
MONO	monocyte
D-D	D-dimer
ALT	alanine aminotransferase
AST	aspartate aminotransferase
ALB	albumin
Scr	serum creatinine
ESR	erythrocyte sedimentation rate
OR	odds ratio
CI	confidence interval
ROC	receiver operating characteristic
AUC	the area under the curve
SD	standard deviation
